# Electronic Cigarette Use during Pregnancy: Is It Harmful?

**DOI:** 10.3390/toxics11030278

**Published:** 2023-03-18

**Authors:** M. J. Ruzmyn Vilcassim, Samuel Stowe, Rachel Majumder, Akila Subramaniam, Rachel G. Sinkey

**Affiliations:** 1 Department of Environmental Health Sciences, School of Public Health, The University of Alabama at Birmingham, Birmingham, AL 35233, USA; gtostowe@uab.edu; 2 School of Health Professions, The University of Alabama at Birmingham, Birmingham, AL 35233, USA; 3 Center for Women’s Reproductive Health, Division of Maternal Fetal Medicine, Heersink School of Medicine, The University of Alabama at Birmingham, Birmingham, AL 35233, USA; asubramaniam@uabmc.edu (A.S.); rsinkey@uabmc.edu (R.G.S.)

**Keywords:** electronic cigarettes, vaping, pregnancy, perinatal health, biomarkers

## Abstract

Although combustible cigarette smoking rates have declined in recent years, alternative tobacco product use, particularly electronic cigarette use (“vaping”), has increased among young adults. Recent studies indicate that vaping during pregnancy is on the rise, possibly due to the perception that it is a safer alternative to combustible cigarette smoking. However, e-cigarette aerosols may contain several newer, potentially toxic compounds, including some known developmental toxicants that may adversely impact both the mother and the fetus. However, there is paucity of studies that have examined the effects of vaping during pregnancy. While the adverse perinatal outcomes of cigarette smoking during pregnancy are well established, the specific risks associated with inhaling vaping aerosols during pregnancy requires more research. In this article, we discuss the existing evidence and knowledge gaps on the risks of vaping during pregnancy. Studies that investigate vaping-associated systemic exposure and its effects (i.e., biomarker analyses) and maternal and neonatal clinical health outcomes are needed to reach more robust conclusions. We particularly emphasize the need to go beyond comparative studies with cigarettes, and advocate for research that objectively evaluates the safety of e-cigarettes and other alternative tobacco products.

## 1. Introduction

As conventional cigarette smoking in the United States (U.S.) has decreased in recent years, the use of alternative tobacco products has become increasingly more popular and is now a major public health concern. The term ”alternative tobacco products” is a broad term that is most often used to describe non-cigarette tobacco products including smokeless tobacco (chewing tobacco, snuff, and snus), hookah water pipes, and electronic cigarettes [1,2,3]. According to data published in the CDC’s *Morbidity and Mortality Weekly Report*, as of 2020, approximately 1.1% of all U.S. adults used pipes (regular pipes, water pipes, or hookahs), 2.3% used smokeless tobacco products, and 3.7% or 9.1 million individuals used e-cigarettes [4]. Thus, current statistics show that electronic cigarettes (e-cigarettes) are by far the most popular alternative tobacco product used in the U.S., which can be attributed to their rapid rise in popularity amongst teens and young adults in recent years [5]. Approximately 2 million middle and high school students reported currently using e-cigarettes in 2021 [6]. As the popularity of e-cigarette use (“vaping”) has rapidly increased amongst young people, the likelihood of women of reproductive age and pregnant women vaping has also increased significantly. Although statistics on vaping during pregnancy are limited, recent studies show that as many as 2.2–7.0% of individuals report using e-cigarettes during pregnancy [7,8,9,10]. These rates are very likely underestimated due to (1) the tendency of underreporting tobacco product use in surveys [11], (2) most hospital intake questionnaires not having specific questions on newer nicotine delivery devices/e-cigarette use, and (3) variations in terminology used to describe vaping, among others. Given that e-cigarettes are fast evolving and contain newer additives, they have the potential to cause unique harm to maternal and fetal health that are largely unknown in the current literature. The perception that vaping is a “risk free” alternative to cigarette smoking can lead to new and/or increased use of electronic nicotine delivery devices during pregnancy. Therefore, it is imperative that more attention is given to this issue and the potential risks of using alternative tobacco products during pregnancy are researched further.

## 2. History of Tobacco Use and Associated Perinatal Outcomes

While studies on e-cigarettes should not be limited to comparative studies with combustible/conventional cigarettes, lessons learned from combustible cigarette smoking do provide a substantial and well-documented background on the risks of using nicotine-containing tobacco products. Prolonged cigarette use is associated with several adverse health outcomes including cardiovascular disease, pulmonary disease, lung cancer, and diabetes mellitus (Table 1) [12,13,14,15,16,17]. These health outcomes are largely attributed to nicotine and other various toxic compounds in combustible cigarettes. Cigarette smoke contains an estimated 5000 chemicals, with more than 60 suspected to be carcinogenic [12,18]. These compounds are thought to cause disruptions in inflammatory pathways that result in certain diseases and carcinogenesis [13]. Nicotine can be particularly harmful to the nervous system, and recent studies indicate that nicotine exposure from tobacco smoke can impair the development of nervous structures, impact neurotransmission, and promote the development of neurodegenerative and cerebrovascular diseases [19]. Nicotine can also result in addictive behavior and increased cigarette use [20].

While the use of cigarettes adversely affects both men and women, there are specific risks associated with smoking while pregnant, as maternal smoking impacts both the mother and the fetus [21,22]. Women of reproductive age who smoke were shown to have high levels of biomarkers of oxidative stress and inflammation, which may influence women’s reproductive health [23]. It has also been documented that cord blood plasma cotinine levels can be similar to that of smoking mothers and cotinine levels in cord serum can be used to distinguish smoking mothers from non-smoking mothers [24,25]. This suggests that cotinine crosses the placenta, which may increase the risk of spontaneous abortions and premature birth, as cotinine stimulates the production of prostaglandin, a uterine contractor [24]. Cotinine has also been detected in breast milk of women who smoke, as well as in women who are exposed to secondhand smoke [26]. Pregnant women who smoke are also at risk of being exposed to heavy metals in cigarette smoke, most notably cadmium, mercury, and lead [26,27].

Neonatal outcomes associated with smoking during pregnancy are also well researched [28]. Aside from physiological effects, behavioral changes have also been observed in children born to mothers who smoked during pregnancy [29]. In children aged 2–3 years old, maternal smoking was associated with over activeness, aggressiveness, and oppositional tendencies [29]. Overall, it has been established that maternal smoking negatively affects maternal physiological, behavioral, and developmental health, as well as neonatal health (Table 1).

**Table 1:** Health effects associated with smoking.

**Adverse Outcomes Associated with Smoking**	**Chemicals**	**References**
Development of a chemical dependence/physical addiction	Nicotine	Wittenberg, Wolfman et al. (2020) [20]
Alteration of glucose homeostasis and increased risk of developing diabetes mellitus	Nicotine	Kondo, Nakano et al. (2019), Maddatu, Anderson-Baucum et al. (2017), [16,17]
Upregulation of inflammatory cytokines	General cigarette smoke	Kondo, Nakano et al. (2019) [16]
Progression of tumor growth and metastasis	General cigarette smoke	Walser, Cui et al. (2008) [15]
Development of Chronic Obstructive Pulmonary Disease (COPD)	General cigarette smoke	Reynolds, Cosio et al. (2006) [14]
Endothelial dysfunction	General cigarette smoke	Kondo, Nakano et al. (2019) [16]
Increased risk of hypertension	General cigarette smoke	Kondo, Nakano et al. (2019) [16]
Increased risk of cardiovascular disease	General cigarette smoke	Kondo, Nakano et al. (2019) [16]
Increased risk of lung cancer	General cigarette smoke	Walser, Cui et al. (2008), Warren and Cummings (2013) [12,15]
**Adverse perinatal outcomes associated with smoking**	**Chemicals**	**References**
Increased maternal cortisol levels resulting in increased stress	Nicotine, general cigarette smoke	Gould, Havard et al. (2020) [30]
Infant cotinine levels reflect maternal cotinine levels	Nicotine	Pichini, Basagaña et al. (2000) [25]
Increased risk of being overweight or obese during childhood	Nicotine	Holbrook (2016) [31]
Increased risk of spontaneous abortion and premature birth	Nicotine, cadmium, lead, general cigarette smoke	Berlin, Heilbronner et al. (2010), Caserta, Graziano et al. (2013), Chelchowska, Ambroszkiewicz et al. (2013), Rzymski, Tomczyk et al. (2015) [24,32,33,34]
High maternal levels of oxidative stress biomarker (F2PG2a) and the inflammation marker (sICAM)	General cigarette smoke	Perez, Mead et al. (2021) [23]
Increased risk of fetus developing neurological, developmental, and endocrine disorders	Cadmium, lead, mercury	Caserta, Graziano et al. (2013) [34]
Increased concentrations of heavy metals in breast milk	General cigarette smoke	Szukalska, Merritt et al. (2021) [26]
Deceased infant systolic blood pressure (SBP)	Manganese, general cigarette smoke	Zhang, Liu et al. (2021) [35]
Decreased infant birth measurements (low birth weight, reduced abdominal circumference, reduced femur length, and reduced head circumference)	Cadmium, lead, general cigarette smoke	Newnham, Patterson et al. (1990), Orlebeke, Knol et al. (1999), Caserta, Graziano et al. (2013), Abraham, Alramadhan et al. (2017), Quelhas, Kompala et al. (2018) [28,29,34,36,37]

## 3. Current Knowledge on the Health Effects of E-Cigarette Use

E-cigarettes are devices that heat an “e-liquid” consisting of propylene glycol or vegetable glycerin, nicotine, and flavoring compounds. E-cigarettes and other Electronic Nicotine Delivery Devices (ENDS) are similar to traditional cigarettes in that both are vessels for delivering nicotine to their users through the inhalation route of exposure. E-cigarettes share several common toxicants with traditional cigarettes, for which the negative health effects of exposure are already well known. However, there are also numerous chemicals found in e-cigarettes that are not found in cigarettes, for which the effects of exposure remain unknown [38]. Furthermore, while the flavorings used in e-cigarettes are approved for oral consumption, they are not approved for inhalation, and when heated, some may undergo changes that could potentially make them more toxic [38,39]. Thus, e-cigarettes are contributing to an entirely new exposure population as more previously unexposed people have begun vaping. Despite this, there is currently a paucity of research on the health effects of exposure to e-cigarette aerosols.

Most of the current research on the health effects of e-cigarettes have focused on comparing them to traditional cigarettes, for the purpose of identifying whether they could be a “safer” alternative to traditional cigarettes [40,41]. While current research does suggest that e-cigarette vapor contains fewer toxic chemicals compared to traditional cigarette smoke [42,43,44] it is important to objectively evaluate the health effects of vaping due to the new and unique compositions of chemicals used in e-cigarettes. Current knowledge indicates that vaping may increase the risk of cardiopulmonary diseases as well as alter immune function [41,44]. This is concerning as vaping has become increasingly more popular among those who have never smoked cigarettes or had nicotine in any other form prior to starting vaping [6,45,46,47].

E-cigarettes heat and vaporize a manufactured e-liquid to provide a “hit” to the user; therefore, they can use one of two different forms of nicotine, freebase nicotine and protonated nicotine/nicotine salts, the latter of which is more potent [41,48]. Freebase nicotine is the traditional form of nicotine found in e-liquids; however, nicotine salts became more popular in e-liquids with the introduction of pod-type devices, such as “JUUL”, because they are more potent and less irritative than freebase nicotine and could therefore be more enjoyably used at higher concentrations [41,48,49,50]. As a result, e-liquids containing nicotine salts deliver a higher internal dose to the user, which is concerning not only because nicotine is addictive and therefore encourages continued use of e-cigarettes and other nicotine containing products, but also because nicotine has been linked to several adverse health outcomes (Table 2) [50,51,52,53,54].

Although still emerging, studies have linked exposure to e-cigarette vapor with several other adverse health effects including altered immune function, cardiovascular inflammation and diseases, respiratory inflammation and illness, increased airway resistance, and chronic respiratory conditions (Table 2) [41,51,54,55,56,57,58,59]. While there is currently a limited amount of research on the cardiovascular effects of e-cigarettes, carbonyl compounds, which are known to adversely impact cardiovascular health, can be found in e-cigarette vapor [57,58]. Studies that have looked at vaping and airway inflammation have indicated that vaping may be associated with acute injury to the small airways and alveoli which may also affect airway clearance [60,61,62,63,64]. However, the greatest example of the degree to which e-cigarette use can affect respiratory health came from the e-cigarette or vaping product use-associated lung injury (EVALI) outbreak that occurred in the United States in 2019. As of February 2020, 2807 cases of EVALI, including 68 deaths, had been reported to the CDC [65]. EVALI patients exhibited acute severe pulmonary illness and often required critical care and respiratory support despite them being otherwise healthy adults [66]. The EVALI outbreak showed that vaping has its own unique risks separate from those associated with smoking that are not currently known or fully understood, and therefore merit research of their own [40,65].

Lastly, while cancer has not been linked with vaping as of yet, many of the volatile organic compounds (VOCs) and heavy metals that have been found in e-cigarette vapor are known carcinogens and therefore pose a threat nonetheless [67,68,69]. The major issue with linking e-cigarette use to cancer is that despite knowing that carcinogens exist within e-cigarette vapor, the timeline to observe an increase in cancer incidence among long-term vapers is unknown. Despite this, there is evidence to suggest that it is biologically plausible that long-term exposure to e-cigarette vapor has the potential to increase one’s cancer risk [68,70]. Furthermore, biomarkers of the carcinogens found in e-cigarette vapor have been identified in higher concentrations in the urine of e-cigarette users than those found in non-e-cigarette using controls [71,72].

**Table 2:** Health effects associated with e-cigarette use (vaping).

**Adverse Outcomes Associated with Vaping**	**Chemical**	**Reference**
Development of a chemical dependence/physical addiction	Nicotine	Marques, Piqueras et al. (2021), Dinardo and Rome (2019) [73,74]
Increased incidence of mental illness	Nicotine	Becker, Arnold et al. (2020) [53]
Altered cardiovascular functioning including increase blood pressure, heart rate, and contractility	Nicotine	Merecz-Sadowska, Sitarek et al. (2020) [51]
Altered glucose homeostasis and increased risk of developing diabetes mellitus	Nicotine	Maddatu, Anderson-Baucum et al. (2017), Kondo, Nakano et al. (2019) [16,17]
Immunosuppression and altered immune function	Nicotine	Gotts, Jordt et al. (2019) [41]
Cardiovascular inflammation	Carbonyl compounds, ultrafine particles	Benowitz and Fraiman (2017), Glantz and Bareham (2018) [57,58]
Endothelial dysfunction	Carbonyl compounds, flavoring compounds	Kennedy, van Schalkwyk et al. (2019) [75]
Increased risk of myocardial infarction	General e-cigarette aerosol	Lippi, Favaloro et al. (2014) [76]
Lung epithelial cell inflammation	General e-cigarette aerosol	Muthumalage, Lamb et al. (2019) [56]
Small airway and alveoli injury	Propylene glycol, glycerol, flavoring compounds, ultrafine particles	Carter, Tucker et al. (2017), Ghosh, Coakley et al. (2018), Reidel, Radicioni et al. (2018), Viswam, Trotter et al. (2018), Chaumont, van de Borne et al. (2019) [60,61,62,63,64]
Increased airway resistance	General e-cigarette aerosol	Honeycutt, Huerne et al. (2022) [55]
Increased incidence of asthma	General e-cigarette aerosol	McConnell, Barrington-Trimis et al. (2017), Schweitzer, Wills et al. (2017) [77,78]
Increased incidence of chronic bronchitis	General e-cigarette aerosol	McConnell, Barrington-Trimis et al. (2017) [77]
EVALI	Vitamin-E acetate, general e-cigarette aerosol	Crotty Alexander, Ware et al. (2020), Krishnasamy, Hallowell et al. (2020) [40,79]

Abbreviations: EVALI: E-cigarette or vaping use-associated lung injury.

## 4. Alternative Tobacco Product Use during Pregnancy and Potential Health Risks

Studies investigating the prevalence of alternative tobacco product use during pregnancy report that less than 1% use smokeless tobacco, 2.5% use hookahs, and 2.2% to 7% of pregnant women use e-cigarettes, with some studies estimating e-cigarette usage to be as high at 15% [7,8,9,10]. The first wave of the Population Assessment of Tobacco and Health (PATH) study revealed that 4.9% of pregnant women use e-cigarettes [8]. The 2015 Pregnancy Risk Assessment Monitoring System (PRAMS) for Oklahoma and Texas reported that the prevalence of vaping around the time of pregnancy was 7.0% overall (10.3% in Oklahoma and 6.5% in Texas) [80] while vaping during the last 3 months of pregnancy was 1.4% (3.2% in Oklahoma and 1.1% in Texas). Among those who vaped, 50–75% reported dual use (i.e., e-cigarettes and combustible cigarettes). Although reported rates vary depending on the sampled populations, they are in general agreement that vaping among pregnant women is on the rise.

Findings further reveal that perceptions greatly influence vaping among pregnant women, despite the unknown risks to maternal and fetal health. Nearly half of the women who vaped in the PRAMS study believed that vaping was less harmful than smoking [80]. Overall, studies on perceptions show two key themes among pregnant women on vaping [7,80,81]: (1) e-cigarettes are safer and a potentially healthier alternative to combustible cigarettes (for the mother and baby) and (2) they may be used as a tool for smoking cessation. Such perceptions combined with a substantial proportion of young women starting vaping at an early age could lead to more women initiating and/or continuing vaping during pregnancy.

In addition to the perception that vaping is safer than smoking, flavorings and other additives in e-cigarettes can be particularly appealing during pregnancy. By removing the smell and sense of tobacco, flavorings make vaping more attractive than combustible cigarette smoking, drawing new users from vulnerable populations [82]. Preferences for sweet flavored e-cigarettes among youth and cigarette smokers trying to quit have been reported [82,83]. Pregnant women may also be vulnerable to the appeal of flavorings due to alterations in taste, cravings, nausea during pregnancy, and other related changes such as an increased sensitivity to bitter tastes during pregnancy [82]. Increased sensitivity to bitter tastes were more likely to lead to the use of menthol cigarettes among pregnant women [84]. Despite evidence for the potential increased susceptibility of pregnant women to flavored products, little is known regarding specific maternal and fetal effects of being exposed to chemicals used in flavorings.

The health risks from tobacco and alternative tobacco product use are even more significant during pregnancy because maternal use impacts both the mother and the fetus. Furthermore, the physiological changes occurring in the cardiovascular and respiratory systems during pregnancy place pregnant patients at a particularly high risk to experience adverse effects from exposure to inhalation toxicants. As discussed above, the detrimental effects of combustible cigarette smoking on perinatal health are well researched and established, demonstrating that it can cause a range of adverse health effects, including low birth weight, preterm birth, neurocognitive and behavioral effects, possibly long-term epigenetic programming, and small for gestational age infants [28,30,85]. Small for gestational age status is of concern due to the Barker Hypothesis that posits a neonate who had stunted intrauterine growth has an increased lifetime risk of cardiovascular and other diseases [86]. E-cigarettes were therefore considered by many as an alternative harm reduction nicotine delivery method during pregnancy. There is, however, growing concern about the increasing use of e-cigarettes and the safety of toxicant exposure for the mother and developing fetus [54].

E-cigarettes share several common toxicants with traditional cigarettes, including nicotine and a variety of volatile organic compounds (VOCs), as well as heavy metals, for which the maternal and fetal health effects of exposure have already been well established (Table 1). Therefore, it is highly possible that exposure to these same compounds via e-cigarette vapor can cause similar impacts, although further research is needed to understand the variations in exposure concentrations and compositions generated from heating of the e-liquids (as opposed to combustion), and the synergistic effects with other unique toxicants in e-cigarette vapor. In the absence of sufficient research on the maternal health impacts of vaping, applying the precautionary principle is advisable, given the potential risks from known compounds. The US Surgeon General’s report on “E-Cigarette Use Among Youth and Young Adults” in 2016 states that “*the effects of nicotine and the potential for harm by other e-cigarette toxicants indicate that the use of ENDS is a fetal risk factor”* [54]. While the existing limited research on vaping-related clinical perinatal outcomes provides some indication of adverse effects, current findings are mixed. Some studies have demonstrated that exclusive vaping during pregnancy did not result in a change in birth weight compared to non-smokers [87], while others have shown that vaping during pregnancy may lead to reductions in birth weight and gestational age and an increase in preterm birth [88,89] Importantly, among e-cigarette users (who did not smoke cigarettes), vaping before pregnancy was not associated with low birth weight or preterm birth compared to non-users [89]. Apart from these outcomes, data on the impact of vaping on other perinatal outcomes are very limited. Animal studies on the effects of fetal exposure to e-cigarette aerosols during pregnancy have provided some evidence of exposure being associated with increases in pro-inflammatory cytokines in the lungs of exposed offspring, altered gene expression and central metabolic expression in offspring, gestational craniofacial and cardiovascular defects, impaired memory, and altered neurodevelopment [90,91,92,93,94,95].

Of interest are other cardiovascular and respiratory outcomes such as asthma, preeclampsia, gestational diabetes, and chronic hypertension (CHTN). However, evaluating some of these health parameters and associations can be complicated and challenging, as conditions such as asthma and CHTN can predate pregnancy. Pregnancy is also a stressful time, associated with depression, and can impact vaping or vice versa. However, studies on the links between depression and vaping among pregnant women are very limited. Rollins et al. [96] observed that pregnant e-cigarette users were more likely to report depression and other severe mental health conditions compared to non-smokers. We are unaware of any other published studies that have analyzed depression and anxiety among pregnant e-cigarette users, which warrants further study.

Analyzing the effects of exposure to complex mixtures of chemicals in e-cigarettes would require both the evaluation of clinical outcomes as well as exposure metabolites and systemic effects. Exposure to xenobiotics results in the production of biomarkers that can be identified in bodily fluids and tissues, constituting biomarkers of exposure. Biomarker studies can demonstrate internal exposure to toxic chemicals associated with tobacco/ENDS use [42,67,97], and elevated levels indicate increased risk of potential of harm [98,99] A major challenge has been the identification and validation of exposure biomarkers specific to e-cigarette use, which is an urgent public health problem. Existing studies have mostly made use of biomarkers developed for smoking, specific to the use of tobacco/nicotine [67,100]. These combustible tobacco-related biomarkers are useful to understand exposures to known chemicals [98]. However, e-cigarettes may result in new exposures. In addition, other less specific biomarkers of tobacco product exposure, such as metabolites of VOCs and polycyclic aromatic hydrocarbons (PAHs), can provide additional information for a more comprehensive exposure assessment to relate exposure to effect and/or outcomes. Studies analyzing exposure biomarkers associated with vaping are emerging, but with very limited studies on pregnant users. Due to the biological changes occurring during pregnancy, the expressions of these biomarkers in pregnant users need to be characterized.

In addition to the knowledge gap on exposure biomarkers in pregnant users, biomarkers of effect associated with vaping during pregnancy have also not been adequately investigated. E-cigarette aerosol exposure has been linked with the expression of inflammatory cytokines from both in vitro and in vivo studies. Exclusive e-cigarette use was associated with elevated serum high-sensitivity C-reactive protein levels and increased expression of inflammatory cytokines [101]. Urinary inflammatory biomarkers were also higher in e-cigarette users compared to non-users [102,103]. However, an analysis of inflammatory and oxidative biomarker concentrations in the PATH study (Population Assessment of Tobacco and Health) did not find a difference between e-cigarette users and non-users [101]. Notwithstanding these findings, contemporary studies characterizing effect biomarkers/inflammatory cytokines in plasma and urine in pregnant users are extremely rare. Due to the other stresses and biological changes occurring during pregnancy, inflammatory biomarker expression during pregnancy may vary from non-pregnant users, and if identified, will be a significant contribution to the knowledge on vaping-induced injury and risks during pregnancy.

While there is a pressing need for studies on the potential risks of vaping during pregnancy, including assessments of exposure and effect biomarkers and perinatal outcomes, conducting such studies can be challenging. Among the many gaps and challenges, the following can be particularly limiting: (1) the paucity of accurate estimates of pregnant patients that exclusively vape due to limited information from hospital electronic medical records (EMRs) on alternative tobacco product use, (2) variations in vaping patterns and frequencies between trimesters, which can lead to exposure variations, (3) dual or multiple use of tobacco products during pregnancy, (4) effect of secondhand exposure from partners, friends, parents, etc., and (5) the limited methods and facilities available to analyze e-cigarette specific biomarkers and the high cost for such analyses. However, these gaps also provide opportunities for new studies and conclusions. Finally, we again emphasize the need to go beyond only comparative studies with cigarettes, and advocate for research that objectively evaluates the safety of alternative tobacco products, especially in the case of pregnant users and other vulnerable groups.

## 5. Conclusions

E-cigarette use has become a major public health concern as prevalence rates among young adults have increased significantly over the past several years. As more young women have begun to vape, there has been an increase in the prevalence of women vaping during pregnancy. Although e-cigarettes have been promoted as a safer alternative to combustible cigarette smoking, vaping aerosols can contain unique toxic compounds, and therefore they cannot be considered objectively safe to use during pregnancy. There is evidence to suggest that exposure to e-cigarettes during pregnancy has the potential to harm maternal and fetal health and cause adverse effects, including increased systemic inflammation, low birth weight, preterm birth, and small size for gestational age status. However, research remains limited and there are large knowledge gaps regarding effects of e-cigarette use on maternal and fetal health and birth outcomes.

## Data Availability

Data sharing not applicable.
